# The effects of enactment on communicative competence in aphasic casual conversation: a functional linguistic perspective

**DOI:** 10.1111/1460-6984.12392

**Published:** 2018-05-14

**Authors:** Rimke Groenewold, Elizabeth Armstrong

**Affiliations:** ^1^ School of Medical and Health Sciences, Edith Cowan University Joondalup, Perth, WA, Australia & Center for Language and Cognition Groningen, University of Groningen The Netherlands; ^2^ School of Medical and Health Sciences Edith Cowan University, Joondalup Perth WA Australia

**Keywords:** aphasia, discourse analysis, speech function analysis, enactment, assertiveness

## Abstract

**Background:**

Previous research has shown that speakers with aphasia rely on enactment more often than non‐brain‐damaged language users. Several studies have been conducted to explain this observed increase, demonstrating that spoken language containing enactment is easier to produce and is more engaging to the conversation partner. This paper describes the effects of the occurrence of enactment in casual conversation involving individuals with aphasia on its level of conversational assertiveness.

**Aims:**

To evaluate whether and to what extent the occurrence of enactment in speech of individuals with aphasia contributes to its conversational assertiveness.

**Methods & Procedures:**

Conversations between a speaker with aphasia and his wife (drawn from AphasiaBank) were analysed in several steps. First, the transcripts were divided into *moves*, and all moves were coded according to the systemic functional linguistics (SFL) framework. Next, all moves were labelled in terms of their level of conversational assertiveness, as defined in the previous literature. Finally, all enactments were identified and their level of conversational assertiveness was compared with that of non‐enactments.

**Outcomes & Results:**

Throughout their conversations, the non‐brain‐damaged speaker was more assertive than the speaker with aphasia. However, the speaker with aphasia produced more enactments than the non‐brain‐damaged speaker. The moves of the speaker with aphasia containing enactment were more assertive than those without enactment.

**Conclusions & Implications:**

The use of enactment in the conversations under study positively affected the level of conversational assertiveness of the speaker with aphasia, a competence that is important for speakers with aphasia because it contributes to their floor time, chances to be heard seriously and degree of control over the conversation topic.

**What this paper adds**

**What is already known on the subject**

Enactment is a device in interaction that allows speakers with aphasia to reveal their communicative competence in a number of ways: it allows them to reduce grammatical complexity, exploit non‐verbal and paralinguistic skills (e.g., shifts in global pitch, volume, tempo, rhythmic patterns and voice quality effects), and get a message across in a vivid, involving way.

**What this paper adds to existing knowledge**

We now know that the use of enactment by speakers with aphasia makes their contributions to conversations more assertive. To our knowledge this is the first research designed to measure conversational assertiveness in casual interaction involving individuals with aphasia. The design is suitable for interaction analysis in both clinical and non‐brain‐damaged populations.

**What are the potential or actual clinical implications of this work?**

Speakers with aphasia suffer from a reduced ability and opportunity to engage fully in conversations. The positive effect of the use of enactment on conversational assertiveness found in this study is in line with outcomes of previous studies indicating that enactment is a device in interaction involving individuals with aphasia that reveals communicative competences that otherwise would remain hidden. Altogether, these findings support a functional therapy approach in which attention is paid to using strategies to compensate for language impairments, and generalization of communication skills in different communicative contexts.

## Introduction

Enactment is a discourse phenomenon wherein a speaker employs direct reported speech and/or other behaviour such as the use of gesture, body movement and/or prosody to depict to recipients aspect(s) of a reported scene or event (Goodwin 1990, Streeck and Knapp 1992, Wilkinson et al. 2010). In conversation, enactment can be used to refer to what someone previously did, said or wrote, but in fact it is more commonly used to communicate thoughts, behaviours or ideas that are prototypical, hypothetical or imaginary, and therefore not reported (Tannen 1986, Semino and Short 2004, Sams 2010, Groenewold et al. 2013). In typical interactions involving non‐brain‐damaged speakers, the occurrence of enactment is pervasive, found across languages and used in diverse contexts (Hengst et al. 2005). It is a natural vehicle for vivid and dramatic presentation (Li 1986), making speech more involving for the listener (Wierzbicka 1974, Redeker 1991, Baynham 1996, Sakita 2002, Tannen 2007) and is often used in stories, jokes and other genres.

Previous research on the occurrence of enactment in interactions involving speakers with aphasia has shown that its use is usually preserved (Ulatowska and Olness 2003, Hengst et al. 2005, Wilkinson et al. 2010, Ulatowska et al. 2011). In fact, studies investigating frequency of occurrence have shown that speakers with aphasia rely on enactment even more often than non‐brain‐damaged language users (Berko Gleason et al. 1980, Groenewold et al. 2013). Possible explanations for this include (1) utterances containing enactment tend to be syntactically less complex (Wilkinson et al. 2010); (2) enactment in everyday interaction is usually heavily marked with paralinguistic resources (e.g., prosody and vocal quality) and non‐linguistic behaviours (e.g., gestures and postures) (Günthner 1999, Hengst et al. 2005), allowing the speaker with aphasia to add information to talk that would otherwise be too complex to put into words; and (3) the occurrence of enactment may be beneficial for speakers with aphasia because it likely facilitates listeners’ language comprehension (Hengst et al. 2005, Groenewold et al. 2014). In other words, enactment could be said to be a device in interaction that allows speakers with aphasia to reveal their communicative competence (Berko Gleason et al. 1980, Hengst et al. 2005, Wilkinson et al. 2010, Ulatowska et al. 2011, Groenewold et al. 2013).

The current study focuses on the effects of enactment on an unexplored but prominent aspect of communicative competence in everyday interaction involving people with aphasia, namely that of *conversational assertiveness*. Conversational assertiveness refers to ‘the capacity to make requests; to actively disagree; to express positive or negative personal rights and feelings; to initiate, maintain, or disengage from conversations; and to stand up for oneself without attacking another’ (Richmond and McCroskey 1985: 92). It becomes manifest in, for example, the capacity to obtain and retain the conversational floor, express knowledge or opinions, or disagree with another speaker (Merrill et al. 2015). These skills are inherently vulnerable in people with aphasia, threatening their opportunities to be active, competent conversation partners and, more generally, their social participation, dramatically disrupting everyday life (Code and Herrmann 2003). Capacities related to conversational assertiveness are therefore essential in people with aphasia's frequently reported desires to recover communicative confidence and be treated as competent conversation partners (e.g., Worrall et al. 2011).

This study investigates to what extent the occurrence of enactment affects the level of conversational assertiveness in everyday interaction involving people with aphasia. In order to assess the level of conversational assertiveness, the framework of systemic functional linguistics (SFL) (Halliday and Matthiesen 2004) will be applied. Whereas aphasia assessment and intervention commonly focus on the identification and treatment of impairments (e.g., deficits in syntax, semantics or phonology), the SFL framework allows for a focus to be made on strengths. This approach reveals insights into how a speaker can convey meanings and the kinds of meanings s/he conveys (Armstrong 2005). In working within an SFL framework, Halliday and Matthiesen (2004) offer a system network to assess the process of positioning co‐interactants into predicted speech roles, reflecting how they adjust their alignments and intimacy with each other. Furthermore, the network allows for an examination of the choices a speaker has in his/her repertoire in terms of initiating or responding, and supporting or confronting the other speaker, as both participants engage each other and maintain the flow of conversation (Armstrong and Mortensen 2006).

Another advantage of the SFL approach is that it addresses the increasingly acknowledged need to look for ways to tap into *real* everyday social interaction in people with aphasia (e.g., Armstrong 2017). To assess discourse skills in people with aphasia, language is typically collected using decontextualized tasks such as picture descriptions, semi‐structured interviews or story retellings (e.g., Dietz and Boyle 2017). While such methods allow for a certain degree of control for form and content of elicited language, the resulting findings may not be indicative of performance in social conversation (Kagan and Gaily 1993, Ross 1999). SFL enables examination of patterns and performance in *authentic* interactions in a systematic way, paying attention to both the speaker with aphasia and the contextual opportunities and constraints that arise during the conversation, and the speaking partner's role in facilitating or impeding the speaker with aphasia's performance (Armstrong and Mortensen 2006, Armstrong et al. 2013). Previous studies applying SFL to interactions involving people with aphasia have proven the suitability of application of the framework to this clinical population (e.g., Armstrong 1992, Ferguson 1992, Ferguson and Elliot 2001, Armstrong 2005, Armstrong and Mortensen 2006, Armstrong et al. 2013, Hersh et al. 2016).

Finally, SFL not only takes into account the kind of social activity that people are engaging in and the interactional process of social communication, but also it allows for a *direct* analysis of conversational assertiveness. Whereas Richmond and McCroskey (1985) relied on self‐assessments to examine assertiveness, the SFL framework allows for examination of conversational data, assessing *actual* rather than *perceived* conversational assertiveness. For the analysis of the current study we will use the framework as discussed by Eggins and Slade (2004). These authors contrast assertive conversational behaviour, where interpersonal relationships are negotiated because positions must be justified or modified, with non‐assertive or deferential behaviour, where an alignment between initiator and ‘supporter’ is created, suggesting a relationship of dependence and subordination (Eggins and Slade 2004). The SFL framework as discussed by these authors can be used to qualify conversational behaviour in terms of level of assertiveness, and reveal the power relationships between interactants throughout conversations. The level of conversational assertiveness is thus a feature of the semantic quality of individual contributions to conversations, and a dynamic process which evolves during interaction. The identification and coding process will be further discussed in the Methods section, and illustrated in the Results section.

Bearing in mind, then, the potential relevance of enactment as a resource to speakers with aphasia, the current study aims to answer the following research question:

- How and to what extent does enactment contribute to conversational assertiveness in everyday interactions involving people with aphasia?

In order to do so, the following sub‐questions will be addressed:

- How assertive are the contributions of individuals with aphasia as compared with those of non‐brain‐damaged individuals in casual conversation?
- How often do individuals with aphasia as compared with non‐brain‐damaged individuals rely on enactment in casual conversation?
- How do enactments affect the level of conversational assertiveness in casual interactions involving speakers with aphasia?

## Materials and methods

### Participants and data

For this explorative case study, we drew on audiovisual data from AphasiaBank, a multimedia database of discourse samples that have been gathered from individuals with aphasia and typical controls (MacWhinney et al. 2011). To examine enactments that speakers with aphasia spontaneously produce in casual interaction, we extracted the largest available data set from the English ‘CA’ (Conversation Analysis) folder on AphasiaBank. This data set was collected, orthographically transcribed and segmented in terms of ‘turns at talk’ (Schegloff 1996) for each participant by Oelschlaeger and Damico (1998). The data consisted of video‐recorded conversations between P, a 50‐year‐old man with a 6‐year history of aphasia, and his wife, M, who had no discernible impairments. P's aphasia quotient (AQ), derived from the administration of the Western Aphasia Battery (WAB), was 46.6 with a WAB classification of conduction aphasia (Oelschlaeger and Damico 1998). The couple made the video recordings in conversational activities and locations of their choosing, representative for types of activities that would occur if the video equipment were absent or if requests for data had not been made. The video camera was placed in the participant couple's home over a 6‐week period, and the couple were asked to turn it on to record their conversations. No schedules or topics were predetermined, and video recording occurred at the couple's discretion (Oelschlaeger and Damico 1998). The original data collection resulted in five two‐party conversations, and three multiparty conversations, where one or two researchers were present. Since the purpose of this study was to examine enactment as it occurs in everyday casual interaction involving individuals with aphasia, we excluded the three multiparty conversations for analysis. The remaining five conversations between P and M took place at their outdoor patio, and lasted between 22 and 53 min, with an overall duration of 174 min.

### Procedures

#### Move identification and coding

As a first step, the turns in the transcripts were transformed into *moves* according to the descriptions and criteria suggested by Eggins and Slade (2004). Although turns are important units in casual conversation, they cannot be used to analyse speech functions, as one turn can realize several functions. Moves are discourse units based on semantic distinctions, i.e., they fulfil a particular function such as agreeing, disagreeing, acknowledging, elaborating. They are typically realized by a clause, but can also be realized by a clause complex in which there is some grammatical dependency between the clauses involved (Halliday and Matthiesen 2004). For example, ‘I think it's great’ or ‘The man was running because he was late’. The end of a move indicates a point of possible turn‐transfer. After a move, a speaker change could occur without turn transfer being seen as an interruption (Eggins and Slade 2004). However, one speaker's ‘turn’ can also consist of several moves. For example, a speaker might make an opening statement, then elaborate further on this statement.

**Figure 1:**
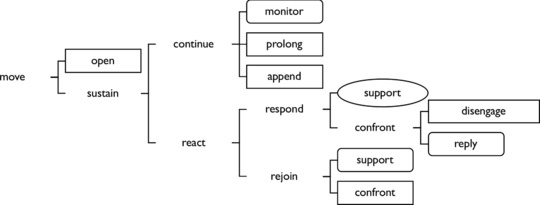
Speech Function Network: Adapted from Eggins & Slade (2004). Assertive moves are presented in rectangles, neutral moves in rounded rectangles, and deferential moves in ovals.

The coding of these moves within the SFL framework reveals patterns of initiating or responding, and supporting or confronting the other speaker, in order to show how interactants explore, adjust, and negotiate alignments and differences. The speech coding framework is comprehensive, in that all moves should be assignable to one of the codes (Eggins and Slade 2004). Once all transcripts were divided into moves, the first author assigned a speech function label to each move (e.g., extract 1). For the coding procedure, the original framework as presented by Eggins and Slade (2004) was used, rather than the simplified version presented in Figure 1. This means that at the first level of delicacy there were two coding options, at the second level there were four options, at the third level there were nine options, at the fourth level there were seven options, and at the fifth level there were 10 options to select from. To check for reliability and accuracy, 10% (*n* = 280) of the moves were re‐coded by the second author. Focusing on the coding of the labels that were of relevance for the distinction between the three assertiveness categories (Figure 1), and to check for interrater reliability, percentage agreement for these labels was calculated. Agreement occurred in 781/958 = 84.3%. This involved levels of delicacy varying from 1 (*n* = 24) to 5 (*n* = 15). Points of disagreement were discussed so that a consensus on the choice of code would be reached.

Extract 1 **(Oelschlaeger and Damico 1998: Scab 8)**

**Table:** 

Turn	ID	Transcription	Coding
484	P:	see, well, Danny was ‐ was good but then he got this &uh meeting and meeting and meeting	Open
486	M:	hmm	Sustain:React:Respond:Support
487	P:	and it's almost like	Sustain:Continue:Append
488	P:	**anything else you want put it in and I'll get to it as soon as I can**	[Open]
489	M:	overworked?	Sustain:React:Rejoin:Support
490	M:	overmeetinged?	Sustain:React:Rejoin:Support
491	P:	meetinged yes	Sustain:React:Rejoin:Support
492	M:	and he can't do his real work for all the meetings	Sustain:React:Rejoin:Support
493	P:	right	Sustain:React:Respond:Support
494	P:	so that's why I says	Sustain:Continue:Prolong
495	P:	**why don't you check on ‐ why don't you hire somebody to check**	[Open]
497	M:	hmm	Sustain:React:Respond:Support
498	P:	and that was a ‐ then we'd go ‐ then we'd go	Sustain:Continue:Append
499	P:	**anything you want**	[Open]
500	M:	right	Sustain:React:Respond:Support
501	P:	but he says	Sustain:Continue:Append
502	P:	**no we'll do it our way**	[Sustain:React:Rejoin:Confront]
503	M:	hmm.	Sustain:React:Respond:Support
504	P:	so…	Sustain:Continue:Append

The data will be presented synoptically through a quantification of the move coding across the interactions for both participants (e.g., Hersh et al. 2016). Furthermore, some extracts will be presented dynamically, illustrating the move coding process, and tracing the move choices as the talk unfolds (Eggins and Slade 2004, see also Ferguson 1992, Ferguson and Elliot 2001, Armstrong and Mortensen 2006, Armstrong et al. 2013, Hersh et al. 2016, Müller and Mok 2012). For the sake of conciseness and easier understanding, we removed all special characters from the original transcript that are not relevant for the current analysis.

The first distinction drawn is between *opening* and *sustaining* moves. Opening moves function to initiate talk around a proposition. This could be through seeking attention, demanding goods or services, asking a question, demanding information, giving information, or providing an evaluation (Eggins and Slade 2004) (e.g., extract 1, line 484).

In *sustaining* moves, the propositions set up in the initiation are being continually negotiated (e.g., extract 1, lines 486, 487). In the case of enactment moves (e.g., extract 1, lines 488, 495, 499, 502), the coding is applied to the enacted communicative acts rather than the current interactional acts.

When the same speaker makes a move, this is classified as a *continuing* move (e.g., extract 1, line 487), which can be further subcategorized into a *monitoring*, a *prolonging* and an *appending* move. *Monitoring* moves imply a readiness to hand over the turn, and seek support for one's own position. *Prolonging* moves enable speakers to flesh out their contributions, getting more than a single move in as speaker. For example, the proposition initiated by P's move in line 484 could have been completed by his agreeing reply in line 493. However, P chooses to use a *prolonging continuing* move (line 494): he prolongs the exchange by adding information to the ongoing negotiation set up in the opening move. *Appending* moves occur when a speaker makes one move, loses the turn, but then as soon as they regain the turn produce a move that is a logical expansion of their immediately prior move (e.g., extract 1, line 487).

When a different speaker makes a move, this is classified as a *reacting* move (e.g., extract 1, line 486). These moves can be further distinguished into *responding* (e.g., extract 1, line 500) and *rejoining* (e.g., extract 1, line 489) moves. *Responding moves* contribute to the completion of the negotiation currently underway, whereas *rejoining* moves delay the completion of the negotiation. Both *responding* and *rejoining* moves can be *supporting* or *confronting. Confronting responding* moves can be further distinguished into *replying* and *disengaging* moves. The further distinctions for *supporting responses*, *supporting rejoinders*, and *confronting rejoinders* will not be reported in the study for reasons of economy of space: the reported level of delicacy depends on whether or not a further distinction between moves is relevant in terms of a better understanding of their level of conversational assertiveness.

### Conversational assertiveness level identification

In the next step of the analysis, we labelled all moves in terms of their level of conversational assertiveness. For this analysis, we relied primarily on the move characteristics as discussed by Eggins and Slade (2004) but also incorporated the definition as provided by Richmond and McCroskey (1985). Below the categorization of all move types in terms of level of conversational assertiveness is discussed. To demonstrate further what different levels of conversational assertiveness look like, in the Results section extracts of the conversations under study will be presented and discussed.

#### Assertive moves

*Opening* moves function to initiate talk around a proposition, and involve a speaker in proposing terms for the interaction. Since they indicate a claim to a degree of control over the interaction, they are considered assertive moves (Eggins and Slade 2004). This characterization is in line with Richmond and McCroskey's (1985) definition which states that the capacities ‘to make requests’ (92), ‘to express positive or negative personal rights and feelings’ (92) and ‘to initiate conversations’ (92) are examples of assertive behaviour in conversation.

Two other types of moves that represent assertive behaviour, are *prolonging* and *appending continuing* moves. Continuing moves enable speakers to flesh out their contribution and keep the turn (Eggins and Slade 2004). This characterization resembles the ‘capacity to maintain conversations’ (92), a skill that Richmond and McCroskey (1985) argue to reflect assertiveness as well.

A fourth move type that was considered assertive, is the *sustain:react:respond:confront:disengage*. The capacity to disengage from a conversation was not discussed by Eggins and Slade (2004) in terms of its assertiveness, but listed as one of the key skills to demonstrate assertiveness in interaction by Richmond and McCroskey (1985).

Finally, according to Eggins and Slade (2004), the most assertive type of move is the *sustain:react:rejoin:confront*. Such a move confronts prior talk by attacking it, by actively rejecting negotiation or by querying the veracity of what has been said. Such moves directly confront the positioning implied in the addressee's move, and hence lead to further talk in which positions have to be justified or modified. This behaviour expresses independence on the part of the speaker, and is therefore highly assertive (Eggins and Slade 2004).

#### Deferential moves

A type of move that is explicitly referred to as non‐assertive by Eggins and Slade (2004) are those labelled *sustain:react:respond:support*. *Supporting replies* expand on previous moves produced by the other conversation partner, agree to the negation going ahead, or indicate a willingness to accept the propositions or proposals of other speakers. They are very co‐operative and minimally negotiatory in nature. Since these moves create an alignment between initiator and supporter but suggest that the relationship is one of dependence and subordination, they are even considered deferential (Eggins and Slade 2004).

#### Neutral moves

The moves of the SFL network that are not considered assertive nor deferential by Eggins and Slade (2004) and Richmond and McCroskey (1985) were considered neutral in terms of their conversational assertiveness. This was the case for *monitoring* moves, which involve moves where speakers focus on the state of the interactive situation, seek support for their own position, or imply a readiness to hand over the turn (Eggins and Slade 2004). The second move type that was considered neutral are those labelled *sustain:react:respond:confront:reply*. These moves encode relatively weak forms of non‐compliance, close off the exchange and avoid overt negotiation of differences (Eggins and Slade 2004). The final move type that was considered neutral in terms of conversational assertiveness, were those labelled *sustain:react:rejoin:support*. Contrary to their challenging *confronting rejoining* counterparts, these moves do not indicate disagreement. They mainly check, confirm, clarify or probe the content of prior moves, and hence delay anticipated exchange completion (Eggins and Slade 2004).

In Figure 1, in a simplified version of the SFL network an overview is presented of the categorization of moves that are considered relevant in terms of their level of conversational assertiveness. As is clear, the depth of the move analysis varied, with continuing moves requiring a less delicate analysis (i.e., three levels) than reacting moves (i.e., four or five levels).

### Enactment identification

Next, we identified all moves in the five conversations that represent enactment. To identify such moves, attention was paid to ‘formal’ criteria such as the occurrence of person references and reporting verbs, but also to prosodic and non‐verbal markers, such as the occurrence of pauses in speech, and shifts in posture, gaze, movement, facial expression, voice quality and pitch height (Lind 2002, Groenewold et al. 2014). To establish interrater reliability, the identification process was carried out by two researchers individually. All identified enactments were compared and discussed. Complex cases were few (*n* = 3) and discussed until agreement was reached. In case of doubt, instances were not labelled enactments. Since enactments reflect aspects of scenes or events that are removed from the current interaction in time or space, their status deviates from the surrounding, typical moves. As enactments insert aspects from past, hypothetical, prototypical, future, or imaginary interactions or events into a current interaction, they can be considered ‘embedded’ moves. To indicate this embeddedness we use brackets (e.g., [enactment]). Related to this embeddedness is their deviating status: Enactments are inherently more independent of surrounding moves since they form part of a (hypothetical) report of a scene or interaction that does not currently take place. As a consequence, they are not elliptically dependent on prior moves, unless they form part of a sequence. The process of identification and analysis of enactment will be illustrated discussing extract 2, and further demonstrated in the Results section.

Extract 2 **(Oelschlaeger and Damico 1998: Scab 2)**

**Table:** 

Turn	ID	Transcription	Coding
541	P:	he is the (gestures:up) way up top	Sustain:React:Respond:Support
542	M:	hmm, right place, right time?	Sustain:React:Rejoin:Support
543	P:	right	Sustain:React:Respond:Support
544	P:	yeah	Sustain:Continue:Prolong
545	P:	that ‐ uh occasionally you'll get say	Sustain:Continue:Prolong
546	P:	**how are you?**	[Open]
547	P:	you know?	Sustain:Continue:Monitor
548	M:	mhm	Sustain:React:Respond:Support
549	P:	oh fine	[Sustain:React:Respond:Support]
550	P:	**um d‐ drafting?**	[Open]
551	P:	xxx?	x
552	P:	**no, no**	[Sustain:React:Respond:Confront:Reply]
553	P:	**there's only two so what the hell?**	[Sustain:React:Rejoin:Confront]
554	M:	yeah	Sustain:React:Respond:Support
555	P:	**you want me or ‐ or the other one?**	[Sustain:React:Rejoin:Confront]
556	M:	what's his name, Steve Caid?	Sustain:React:Rejoin:Support

The stretch of talk presented in extract 2 is preceded by comments by P about how people generally find it difficult to deal with P's condition, followed by a question raised by M, asking how Johnson (P's manager) is doing. In line 541, P gives an initial answer, which gets clarified by M (line 542). P then agrees with M and elaborates further in lines 543–545. In line 546, P ascribes a prototypical, recurrent (indicated by the adverb ‘occasionally’, line 545) quote to his manager. Introducing the enactment using the generic ‘you’ in line 545 (as opposed to ‘I’), P stresses the generic nature of the enactment, and the fact that he does not report an actual interaction. The question–answer sequence reflects the power difference P experiences: the manager is senior and therefore the person asking questions. After monitoring (line 547) that M has understood and M's supportive encouragement for P to take another turn (line 548), he produces another enactment (line 549), this time representing speech in the hypothetical situation. Note that P does not explicitly indicate the speaker shift using a pronoun or reporting verb, but uses a discourse marker (‘oh’) instead to indicate this move represents an answer to the prototypical question posed by the hypothetical conversation partner. In line 550, P proceeds ‘reporting’ the hypothetical dialogue, shifting back to the manager, who asks P a closed, factual question. The question underlines the difference in hierarchy: the manager asks whether P is drafting. P used to be an engineer, but as a consequence of his stroke he is carrying out drafts work now. After producing an unintelligible move in 551, in line 552 P reports a hypothetical (disagreeing) answer to the manager's question. Next, in line 553, he expresses a hypothetical confrontation with his manager, offering a counter‐interpretation of the situation raised by the manager in the imaginary conversation (i.e., P being a draftsman rather than an engineer), followed by a hypothetical clarification in line 555.

### Exploration of relationship between enactment and conversational assertiveness

Finally, we examined the co‐occurrence of enactment and conversational assertiveness. To assess whether there is a relationship between the occurrence of enactment and the level of conversational assertiveness, we compared the distribution over the three levels of conversational assertiveness (assertive, neutral, and deferential) between enactments and non‐enactments for both speakers, using a two‐tailed Fisher's exact test. Furthermore, to examine the differential levels of conversational assertiveness between enactments and non‐enactments in a more qualitative way, the distribution of enactments and non‐enactments over assertive move categories will be explored.

## Results

### Move identification and coding

The move identification process resulted in a data set consisting of 2811 moves. P produced 1242 moves during the five analysed conversations, and P's wife M produced 1569 moves. The distributions over move categories for both interactants are presented in Table 1. As is clear, the most frequently occurring move type for P is the *supporting response* (*n* = 430, 35%) (generally considered to be a non‐assertive move type; Eggins and Slade 2004), and that for M is the *prolonging continuer* (*n* = 550, 35%) (considered to be more an assertive move). This pattern is illustrated in the stretch of talk presented in extract 3. P produces several instances of *supporting replies*. In line 60, he *agrees* with M's move, in line 63 he *acknowledges* what M just said, and in lines 66 and 70 he *registers* M's immediately preceding moves. Extract 3 also reflects M's frequent use of *prolonging continuers*: in line 67, clarifying her previous proposition she *elaborates*, and in lines 68 and 69, adding further non‐attitudinal information she *extends* her own moves.

**Table 1:** Distributions over move categories of all speech produced by P and M

	Speaker ID	
Move label	P	M
Open	155	145
Sustain:Continue:Monitor	15	16
Sustain:Continue:Prolong	210	550
Sustain:Continue:Append	98	132
Sustain:React:Respond:Support	430	358
Sustain:React:Respond:Confront:Reply	98	22
Sustain:React:Respond:Confront:Disengage	12	1
Sustain:React:Rejoin:Support	155	297
Sustain:React:Rejoin:Confront	69	48
Total number of moves	1242	1569

Extract 3 **(Oelschlaeger and Damico 1998: Scab 2)**

**Table:** 

Turn	ID	Transcription	Coding
59	M:	you're gonna cut the grass	Open
60	P:	um, yes	Sustain:React:Respond:Support
61	P:	and then I'll go to the Radio Shack and we'll see what they	Sustain:Continue:Prolong
62	M:	what they've got	Sustain:React:Respond:Support
63	P:	got	Sustain:React:Respond:Support
64	P:	and that's about it	Sustain:Continue:Prolong
65	M:	I wanna go to the book store	Open
66	P:	oh	Sustain:React:Respond:Support
67	M:	there's a book on tape I think	Sustain:Continue:Prolong
68	M:	when I was with Jeannie, we went to the used book store	Sustain:Continue:Prolong
69	M:	the lady there had some new books but she didn't do books on tape	Sustain:Continue:Prolong
70	P:	hmm	Sustain:React:Respond:Support

### Conversational assertiveness‐level identification

**Figure 2:**
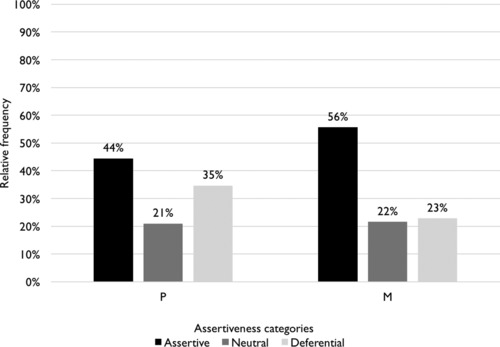
Distribution of moves over assertiveness categories for both speakers.

Figure 2 presents an overview of the moves that represent assertive, neutral, and deferential conversational behaviour by both participants. As is clear, M produces relatively more assertive moves than P. However, the distribution of moves over the three conversational assertiveness categories is similar for P and M: for both speakers, the assertive move category is larger than the other two categories. Around one‐third of all moves produced by P can be considered deferential, and around one‐fifth of all moves are neither assertive nor deferential. The non‐assertive moves produced by M are divided nearly equally over the latter categories.

To demonstrate what the data and different types of conversational behaviour looked like, below we present and discuss two extracts of two of the analysed conversations. Extract 4 presents a stretch of talk in which P exclusively presents assertive conversational behaviour: In line 516, P shows *confronting* conversational behaviour, *challenging* M's immediately prior move. Next, he *prolongs* his previous move (line 517), followed by an (embedded) *opening* move in line 518.

Extract 4 **(Oelschlaeger and Damico 1998: Scab 4)**

**Table:** 

Turn	ID	Transcription	Coding
515	M:	this working is cramping my life style	Sustain:Continue:Append
516	P:	yeah but you know some of these days good and other days is bad	Sustain:React:Rejoin:Confront
517	P:	they uh mostly it's kinda like	Sustain:Continue:Prolong
518	P:	**well I don't want it, you know**	[Open]
519	M:	mhm	Sustain:React:Respond:Support

By contrast, P's conversational behaviour in extract 5 is exclusively deferential. In this passage, he only demonstrates *supportive* conversational behaviour, *replying* to (lines 35, 44, and 46) and *registering* (line 40) M's moves. M's conversational behaviour in this extract can be considered a mixture of deferential (lines 33, 36 and 45) and assertive (lines 39, 41–43) behaviour.

Extract 5 **(Oelschlaeger and Damico 1998: Scab 7)**

**Table:** 

Turn	ID	Transcription	Coding
33	M:	yeah I mean the whole thi(ng) ‐ yeah the whole thing needs to be replaced	Sustain:React:Respond:Support
35	P:	right	Sustain:React:Respond:Support
36	M:	I'm just trying to find out what the stupid things are	Sustain:React:Respond:Support
37	M:	one seventy one	(reads aloud)
38	M:	hmm no	Sustain:React:Respond:Confront:Reply
39	M:	it's not a strap and it's not a tie down	Sustain:Continue:Prolong
40	P:	hmm	Sustain:React:Respond:Support
41	M:	what else would you call it?	Open
42	M:	or it's not in the book	Sustain:Continue:Prolong
43	M:	whatever	Sustain:Continue:Prolong
44	P:	well I'll see (takes catalog)	Sustain:React:Respond:Support
45	M:	I mean (be)cause this is the way they're made	Sustain:React:Respond:Support
46	P:	yeah, that's true	Sustain:React:Respond:Support

### Enactment identification

The enactment identification process resulted in a collection of 79 enactments. Fifty‐eight of these were produced by P (≈ 5% of his moves), and 21 by M (≈ 1% of her moves). To illustrate the procedure of analysis and demonstrate some interesting observations, extract 2 is further discussed here.

Extract 2 **(Oelschlaeger and Damico 1998: Scab 2)**

**Table:** 

Turn	ID	Transcription	Coding
541	P:	he is the (gestures:up) way up top	Sustain:React:Respond:Support
542	M:	hmm, right place, right time?	Sustain:React:Rejoin:Support
543	P:	right	Sustain:React:Respond:Support
544	P:	yeah	Sustain:Continue:Prolong
545	P:	that ‐ uh occasionally you'll get say	Sustain:Continue:Prolong
546	P:	**how are you?**	[Open]
547	P:	you know?	Sustain:Continue:Monitor
548	M:	mhm	Sustain:React:Respond:Support
549	P:	**oh fine**	[Sustain:React:Respond:Support]
550	P:	**um d‐ drafting?**	[Open]
551	P:	xxx?	x
552	P:	**no, no**	[Sustain:React:Respond:Confront:Reply]
553	P:	**there's only two so what the hell?**	[Sustain:React:Rejoin:Confront]
554	M:	yeah	Sustain:React:Respond:Support
555	P:	**you want me or ‐ or the other one?**	[Sustain:React:Rejoin:Confront]
556	M:	what's his name, Steve Caid?	Sustain:React:Rejoin:Support
557	M:	whatever happened to him?	Open

This extract contains a cluster of enactments (indicated in bold) produced by P. Some of the characteristics in this extract are representative for the majority of the enactments produced by P. First, there are no person references. P shifts between speakers (namely his manager and himself) a couple of times, but does not rely on person references to indicate these shifts. Second, P does not use reporting verbs, but instead relies on other markers to indicate enactment (i.e., ‘you'll get say’ in line 545 and ‘oh’ in line 549). None of the enactments refers to a unique speech event: they all represent hypothetical speech. Third, the enactments all form part of question–answer sequences: they are either interrogatives (lines 546, 550, 553 and 555) or answers (lines 549 and 552). Finally, despite the lack of explicit markers and the conciseness of the verbal information provided by P, the enactments do not seem problematic to understand for M: her registering moves (lines 548 and 554) and clarifying question (line 556) show comprehension and encouragement for P to continue.

### Exploration of the relationship between enactment and conversational assertiveness

**Figure 3:**
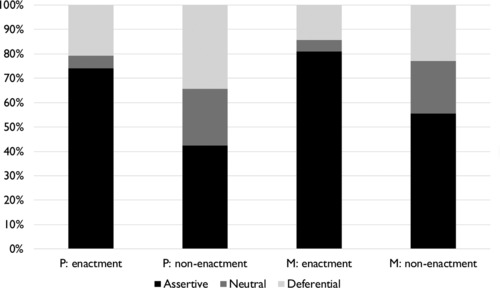
Distribution over assertiveness categories for enactments and non‐enactments produced by both speakers.

Figure 3 presents the relative frequencies for assertive, neutral and deferential types of conversational behaviour for P's and M's enactments and non‐enactments. As is clear, it is more common for P's enactments (*n* = 43/58) than for P's non‐enactments (*n* = 501/1184) to represent assertive conversational behaviour. In addition, deferential conversational behaviour is realized relatively more frequently through non‐enactments (*n* = 406/1184) than through enactments (*n* = 12/58). Statistical analysis (two‐sided Fisher's exact test) confirmed that for P's moves there is a relationship between the occurrence of enactment and the level of conversational assertiveness (*p* < 0.001). No such relationship exists between the presence of enactment and the level of conversational assertiveness for M's moves (*p* > 0.05). The counts used in the Fisher's tests are presented in Table 2.

**Table 2:** Counts of assertive, neutral, and deferential enactment and non‐enactment moves used for Fisher's test

Speaker			Assertive	Neutral	Deferential	Total
**P**	Non‐enactment	Count	501	277	406	1184
		%	42.3%	23.4%	34.3%	100.0%
	Enactment	Count	43	3	12	58
		%	74.1%	5.2%	20.7%	100.0%
	Total	Count	544	280	418	1242
		%	43.8%	22.5%	33.7%	100.0%
**M**	Non‐enactment	Count	859	334	355	1548
		%	55.5%	21.6%	22.9%	100.0%
	Enactment	Count	17	1	3	21
		%	81.0%	4.8%	14.3%	100.0%
	Total	Count	876	335	358	1569
		%	55.8%	21.4%	22.8%	100.0%
**Total**	Non‐enactment	Count	1360	611	761	2732
		%	49.8%	22.4%	27.9%	100.0%
	Enactment	Count	60	4	15	79
		%	75.9%	5.1%	19.0%	100.0%
	Total	Count	1420	615	776	2811
		%	50.5%	21.9%	27.6%	100.0%

To examine the differential levels of conversational assertiveness between enactments and non‐enactments in a more qualitative way, Table 3 presents the distributions over assertive move categories for P's enactments and non‐enactments. As is clear, assertive moves are distributed differently over move categories for enactments and non‐enactments. Assertive enactments are mostly labelled *opening* moves, whereas the largest move category for assertive non‐enactments is the *prolonging continuer*. Extracts of the examined conversations containing assertive enactments are presented and discussed below.

**Table 3:** Distribution over categories of assertive moves produced by P

Move label	Enactment (*n* = 43)	Non‐enactment (*n* = 501)
Open	72.1%	24.8%
Sustain:Continue:Prolong	9.3%	41.1%
Sustain:Continue:Append	7.0%	19.0%
Sustain:React:Rejoin:Confront	9.3%	13.0%
Sustain:React:Respond:Confront:Disengage	2.3%	2.2%

In extract 6, P ‘reports’ thought. There are several indicators that mark the shift in modality indicating the enactment produced in line 167. First, the interrogative is preceded by a verb of cognition (i.e., *think*, which is often used to mark an upcoming ‘report’ of a thought, opinion, attitude or state of mind). Second, the interrogative is introduced by the discourse marker ‘well’. Third, P shifts from ‘I’ (line 166) to ‘we’ (line 167) for person reference. Finally, the enactment is preceded by a pause and marked by a shift in pitch and volume. Following the SFL framework, this closed question is labelled an *opening* move. The way this move indicates a claim to a degree of control over the interaction is twofold: First, posing a question is a way to propose terms for the interaction (Eggins and Slade 2004). Second, posing the question in an embedded (namely enacted) way, P keeps control over this interactional sequence. Before other talk can be initiated, the embedded question needs to be answered. The answer can only be provided by P, who therefore created a situation that pushes for *supporting* (i.e., deferential) behaviour by M. Indeed, M produces a minimal response in line 168, returning the floor to P, who then produces an *appending* move. In other words, whereas closed questions normally present a complete proposition for the support or confrontation of the addressee, P in this abstract secures floor‐time.

Extract 6 **Assertive enactment (Oelschlaeger and Damico 1998: Scab 8)**

**Table:** 

Turn	ID	Transcription	Coding
164	M:	but ‐ a half of ‐ a half of soda box is what you want	Sustain:Continue:Prolong
165	P:	right	Sustain:React:Respond:Support
166	P:	so I think about this and I think	Sustain:Continue:Prolong
167	P:	**well, do we want to or not?**	[Open]
168	M:	hmm	Sustain:React:Respond:Support
169	P:	and mostly I nink (:think) not	Sustain:Continue:Append

Extract 7 is preceded by some talk about one of P's colleagues and her son (referred to as ‘the boy’ in line 372). In line 374, P wants to make a claim about the daughter, using ‘but’ to indicate the contrast in behaviour between her and the boy (who ‘is okay’). P abandons the move without further specifying the girl's behaviour, and in line 375 M shows supportive behaviour for P to continue. After a move that does not further specify the behaviour (line 376), in line 377 M makes an ‘educated guess’, offering further details for confirmation by P. In line 379, P indicates agreement to a certain extent, and after M's supporting response in line 380 P enacts the girl's behaviour rather than describing it. This shift from a describing to an enacting mode is marked in several ways: First, it is introduced by the adverb ‘occasionally’. Second, P produces a pronoun and a reporting verb. These characteristics make the enactment appear as a prototypical enactment. However, typical speakers would probably not use enactment here as it does not concern prominent information, a part of an event line, or the climax of a story (Labov 1972, Mayes 1990). Instead, the information refers to a state of affairs that causes P's colleague to be upset every now and then. Relying on enactment here, however, allows P to maintain control over the interaction, and specify the girl's behaviour rather than being dependent on M volunteering further specifications for confirmation. M's *supporting response* (line 383), her verification of her understanding (line 384) and P's confirmation thereof (line 385) demonstrate the enactment successfully conveyed the intended message.

Extract 7 **Assertive enactment (Oelschlaeger and Damico 1998: Scab 2)**

**Table:** 

Turn	ID	Transcription	Coding
372	P:	the the boy is okay	Sustain:Continue:Prolong
373	M:	uhhuh	Sustain:React:Respond:Support
374	P:	but the girl	Sustain:Continue:Append
375	M:	girl	Sustain:React:Respond:Support
376	P:	is	Sustain:Continue:Append
377	M:	a runaway?	Sustain:React:Rejoin:Support
378	P:	yeah	Sustain:React:Respond:Support
379	P:	well, almost	Sustain:Continue:Prolong
380	M:	hmm	Sustain:React:Respond:Support
381	P:	occasionally she says	Sustain:Continue:Prolong
382	P:	**hi, I'm home!**	[Open]
383	M:	hmm	Sustain:React:Respond:Support
384	M:	one of those kids you wouldn't want for your own	Sustain:Continue:Prolong
385	P:	mhm	Sustain:React:Respond:Support

A similar form and a similar function of enactment are observed in extract 2 (presented in the section Enactment identification). The first enactment in this extract (*you'll get say ‘how are you’*) is an embedded opening move as well, used to specify someone else's attitude/behaviour rather than a specific event. Like the enactment in extract 7, the generic nature of this enactment is marked by the adverb ‘occasionally’. In extract 2, the adverb is followed by the just as generic ‘you’. The purpose of this enactment is not to report a statement, but to demonstrate his manager's attitude, and hence does not typically involve enactment. Two other enactments in extract 2 that are considered assertive are labelled *challenging confronting rejoinders*. In line 553 (*there's only two so what the hell?*), P relies on enactment to demonstrate his opinion. Shifting to a hypothetical world, where he was talking to his manager and dared to express his true opinion, he is able to successfully get his message across, and demonstrate an assertive conversational skill. The same goes for the enactment produced in line 555 (*you want me or—or the other one?*): the challenging nature of this question is only appropriate here because it is part of an enacted (hypothetical) dialogue.

## Discussion and conclusions

The aim of the current study was to shed new light on individuals with aphasia's motives to rely more heavily on enactment than non‐brain‐damaged speakers in naturally occurring interaction (Groenewold et al. 2013). More specifically, we focused on its potential relation with conversational assertiveness.

Previous studies have shown that the frequent occurrence of enactment by speakers with aphasia may reveal communicative competence in a number of ways: it enables them to use grammatically less complex constructions, make optimal use of non‐verbal and paralinguistic skills (such as gestures, shifts in body posture, facial expression, pitch, volume, intonation, and voice quality), and get their message across in a vivid and involving way (Berko Gleason et al. 1980, Hengst et al. 2005, Wilkinson et al. 2010, Ulatowska et al. 2011, Groenewold et al. 2014). In the current study, an additional possible explanation for the increased use of enactment in interaction involving an individual with aphasia is addressed, namely that of its possible beneficial effects on conversational assertiveness.

The first question addressed the general levels of conversational assertiveness for both speakers throughout the conversations under study. The speaker with aphasia produced less assertive moves than his non‐brain‐damaged partner. This outcome is in line with previous literature, reporting a reduced ability and opportunity in speakers with aphasia to engage fully in conversations (Kagan 1995, Ross et al. 2006). This opportunity to be an active conversation partner is essential in people with aphasia's desire to recover communicative competence (e.g., Worrall et al. 2011).

The difference between assertive and deferential moves was also less pronounced in the speaker with aphasia than in the non‐brain‐damaged speaker. In addition to these quantitative differences between the speaker with aphasia and the non‐brain‐damaged speaker, a closer look at the subtypes of contributions within the assertive behaviour category revealed that their moves are distributed differently as well: Whereas the non‐brain‐damaged speaker mainly relies on one specific move type, the assertive moves produced by the speaker with aphasia are distributed more equally over the assertive subcategories. This means that there is not only a quantitative, but also a qualitative difference between the two speakers when it comes to conversational assertiveness. Of course, there may be other factors that contribute to the inequality between the speaker with aphasia and his wife: it may for example be the case that the level of conversational assertiveness has always been unequal for this couple. This potential factor applies to most interactional studies around communicative skills and opportunities involving people with aphasia: if there are no ‘pre‐onset’ conversational data available this is difficult to verify. Importantly, our central research question is focused around the differential effects of enactments and non‐enactments on the level of conversational assertiveness within the speaker with aphasia. The answer to this question is not affected by other, potentially confounding, factors.

The second question addressed the occurrence of enactment throughout the conversations. In line with previous studies (Berko Gleason et al. 1980, Groenewold et al. 2013), both the speaker with aphasia and the non‐brain‐damaged conversation partner were found to produce enactments, with the speaker with aphasia relying on enactment relatively more often than the typical speaker.

The third question assessed the possible relationship between the occurrence of enactment and the level of conversational assertiveness. A shift was observed when comparing the moves that contained enactment to those that did not contain enactment: Of the moves containing enactment, the majority were assertive in nature, whereas of the moves without enactment less than half were assertive. In other words, the occurrence of enactments positively affected the level of conversational assertiveness in the speaker with aphasia's contribution to the examined interactions. Focusing on all assertive moves produced by the speaker with aphasia, not only a quantitative but also a qualitative difference was observed between enactments and non‐enactments. Whereas the most common label for assertive non‐enactments is the *prolonging continuer*, most assertive enactments consist of *opening* moves. This means that assertive non‐enactments mainly keep negotiating the same proposition, whereas assertive enactments often function to initiate talk around a new proposition (Eggins and Slade 2004), and therefore may even be considered more assertive in nature.

The outcomes of the current study are in line with findings of previous studies which indicate that enactment is a device in interaction that allows individuals with aphasia to achieve a range of communicative and semiotic acts that would otherwise be very difficult, resonating Holland's axiomatic suggestion that speakers with aphasia ‘communicate better than they talk’ (Holland 1977: 173). The comparison between enactments and non‐enactments revealed that enactments are generally more assertive in nature than non‐enactments. The qualitative analyses of the enactments showed that enactment is a device in interaction that may help a speaker with aphasia keep control over an interactional sequence eliciting supporting behaviour of the conversation partner, and hence securing floor‐time. Furthermore, the shift to a hypothetical or fictive scenario or dialogue can allow a speaker with aphasia to demonstrate the capacity to produce *challenging confronting* conversational behaviour, a skill that probably will not be demonstrated in a descriptive type of speech.

### Benefits, limitations and future work

The SFL framework used in the current study proved a valuable tool to examine language in ‘real’ interaction in a comprehensive and detailed way, while considering the effects of both lexical and syntactic limitations (Armstrong and Mortensen 2006, Müller and Mok 2012). Importantly, the framework used here has offered an exhaustive scheme for quantification of conversation. Unlike many other scoring systems, which usually measure the contribution of individuals with aphasia in terms of amount of speech rather than in terms of the quality of their contributions, SFL links linguistic form to a system of communicative functions, highlighting the strengths rather than the deficits or constraints of people with aphasia (Müller and Mok 2012).

Some limitations of this study need acknowledgment as well. First, the number of enactments occurring in the data set was limited. Even though enactment was employed throughout the conversations by both speakers, the frequency of occurrence is relatively low when compared with other studies that have found it is commonly employed in around 10% of spoken language (e.g., Johnstone 1993). The low percentage of enactments in the current study is partly due to the fact that one specific type of enactment (namely onomatopoeia, which were produced by both speakers throughout the conversations) had to be removed from the analysis. In order to be attributable to one of the SFL categories, an understanding of the meaning of a move is required, and this is not easily the case for enactments in the form of onomatopoeia. More research on the interactional functions of onomatopoeia can provide guidelines for classification of this special form of enactment. Second, even though it can be considered a strength that the SFL framework is comprehensive, as demonstrated by various other studies (e.g., Armstrong 1992, 2005, Ferguson 1992, Ferguson and Elliot 2001, Armstrong and Mortensen 2006, Armstrong et al. 2013, Hersh et al. 2016), the quantitative character of the framework necessarily elides qualitative nuances in conversational practices. The qualitative description allowed for a more detailed discussion of the data, but was only carried out for a selection of the materials. Related to this, one‐third limitation of the study had to do with the conversational assertiveness categorization system, which did not account for all possible variety in level of assertiveness. Since only three levels of conversational assertiveness were distinguished (i.e., assertive, neutral, and deferential), the method of the current study does not account for the possible existence of a continuum between assertive and deferential contributions to conversations, with neutral somewhere in between. This limitation can be addressed in future research, using a classification system that allows for a more nuanced analysis of conversational assertiveness.

### Suggestions for clinical implications

Even though the results of the current study are not generalizable, it has some potential clinical implications. It has shown that the use of enactment by a speaker with aphasia positively affects assertiveness in casual conversation. In line with the literature, this means there is an important difference between linguistic and communication skills (Holland 1977). Some individuals with aphasia have been shown to benefit from the use of compensatory communication strategies, such as the use of very general or less accurate words that are easy to produce without errors (Laakso 2003), the use of reported speech to circumvent word‐finding or syntactic difficulties (Berko Gleason et al. 1980) or repetition to delay word finding (Armstrong and Ulatowska 2007). Simmons‐Mackie and Damico (1997) defined a compensatory strategy in aphasia as ‘a new or expanded communicative behaviour, often spontaneously acquired and systematically employed, to overcome a communication barrier in an effort to meet both transactional and interactional communicative goals’ (770). The increase in the occurrence of enactment in interaction of speakers with aphasia that was examined in previous research (Groenewold et al. 2013) and the current study meets the criteria of this definition, and may therefore be considered a compensatory communication strategy in aphasia. Therefore, the current findings support a functional therapy approach, in which attention is paid to teaching strategies to compensate for language impairments, and generalization of communication skills and strategies in different communicative contexts. It is possible that enactment may be exploited in expanding a person with aphasia's communicative repertoire, and that it could be encouraged by communication partners once its function and potential for expressing a wider variety of thoughts and opinions is more clearly understood by partners. However, more research is needed to set feasible therapy goals. Depending on the results of these studies, speakers with aphasia who employ enactment in casual conversation may be encouraged to keep doing so, and individuals with aphasia who do not rely on enactment could be encouraged to do so.

### Conclusions

The outcomes of the current study suggest that the increased use of enactment by speakers with aphasia positively affects conversational assertiveness, a competence that is highly relevant for speakers with aphasia because it contributes to their floor time, chances to be heard seriously, and degree of control over the conversation topic (Eggins and Slade 2004).
